# A Nordic initiative for a more personal and accurate diagnostic pathway for prostate cancer

**DOI:** 10.1080/02813432.2020.1801148

**Published:** 2020-08-14

**Authors:** Svein R. Kjosavik, Karina Dalsgaard Sørensen, Kristina Hotakainen, Henrik Grönberg

**Affiliations:** a The General Practice and Care Coordination Research Group, Stavanger University Hospital, Stavanger, Norway; b Faculty of Health Sciences, University of Stavanger, Stavanger, Norway; c Department of Clinical Medicine, Aarhus University, Aarhus, Denmark; d Department of Molecular Medicine, Aarhus University Hospital, Aarhus, Denmark; e Department of Clinical Chemistry, University of Helsinki, Helsinki, Finland; f Mehiläinen Oy Laboratory, Helsinki, Finland; g Department of Medical Epidemiology and Biostatistics, Karolinska Institutet, Stockholm, Sweden; h Department of Oncology, Capio S:t Görans Hospital, Stockholm, Sweden

**Figure 1.**
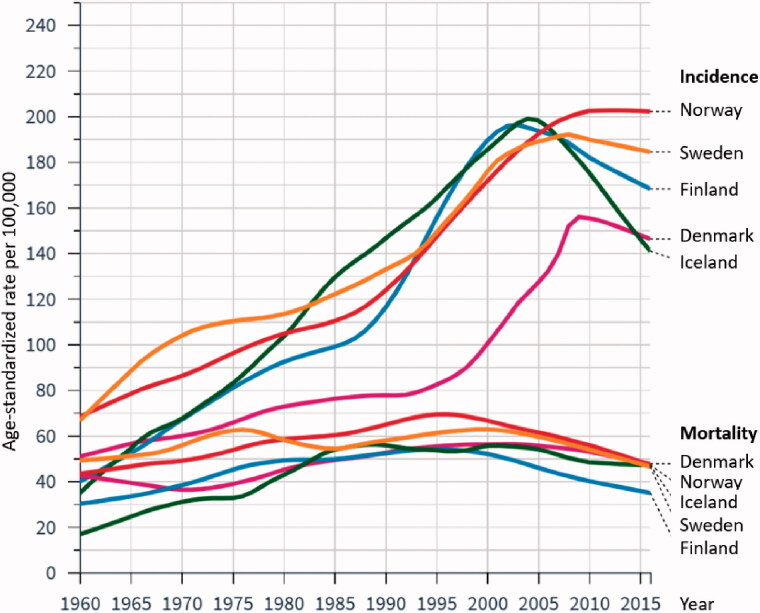
Incidence and mortality rates for prostate cancer per 100,000 inhabitants in the Nordic countries from 1960 to 2016 according to NORDCAN [1].

Prostate cancer (PCa) is currently a textbook example of over-diagnosis and over-treatment. Nevertheless, it is the most commonly diagnosed cancer and the second leading cause of cancer-related death in men in the Nordic countries (Figure 1) [1]. Due to demographic changes, the incidence of PCa is expected to rise by up to 40% in the next 20 years. Consequently, PCa is a challenge for the health care system and health economy.

According to Beauchamp and Childress [2], there are four global ethical principles in medicine: (A) beneficence, (B) non-maleficence, (C) justice and (D) respect of (the patient’s) autonomy. From our perspective, the current diagnostic pathway for PCa challenges all of these principles. First, there is no beneficence for patients in over-diagnosis and over-treatment, but rather a risk of harm. Second, resources in the health care system are not utilized justly. Third, the diagnostic procedure may lack the patient’s consent or, where consent is obtained, it may be uninformed. Consequently, the challenges in dealing with PCa are also ethical.

Under- and over-diagnosis depend on the sensitivity and specificity of the diagnostic tests used. If the sensitivity of a test increases, the specificity usually reduces, and vice versa. Consequently, new tests with higher accuracy are needed to improve the diagnosis of PCa. Henrik Grönberg and his team in Stockholm have been working on a new approach for PCa diagnosis since 2010. Their efforts have resulted in a Nordic research collaboration (NorDCaP) focusing on improving several of the steps in the diagnostic pathway for PCa.

The first and crucial step is to decide whether or not to test. A Norwegian study indicates that GPs in Norway are experiencing strong pressure from patients to conduct examinations to detect illness [3]. While urological symptoms are common [4], their association to PCa is low [5]. The main problem with the test currently in use, prostate-spesific antigen (PSA), is low specificity at a level of acceptable sensitivity. This is why Nordic guidelines recommend against PSA-based population screening, as the problems with over-diagnosis and over-treatment are considered to outweigh the possible benefits. Nevertheless, opportunistic PSA testing is still widespread in the Nordic countries.

Various guidelines state that patients must be well informed before consenting to PCa testing. To improve this process, Capio S:t Göran Hospital in Stockholm has developed and implemented a strategy with mandatory information provision to ensure patients are well-informed before making the decision [6].

The next step is selecting patients for further work-up. To improve this, Grönberg et al. have developed a new blood-based test called Stockholm3. In it, an algorithm combines the results of PSA, four other plasma proteins, more than 100 genetic markers and some clinical data to make a PCa risk assessment. It results in a better selection of men for further work-up than PSA alone [7]. The Stockholm3 test is more expensive than PSA and should be evaluated based on clinical outcome and health economics. Experiences from implementing this test in regular clinical practice in the Stavanger region of Norway are described in a research article in this issue of SJPHC [8].

When men are referred for further diagnostic work-up due to suspected PCa, a prostate gland biopsy is crucial for diagnosis but traditionally only a small proportion of biopsied men are diagnosed with clinically significant PCa; most diagnoses are for clinically non-significant PCa or no cancer at all [9]. Unfortunately, a biopsy results in sepsis among 2–5% of the cases. Consequently, better procedures are needed for selecting biopsied patients. Besides a better blood test, a prostate MRI may be helpful, but there is still debate as to whether the accuracy is sufficient that a biopsy can be avoided in cases of negative MRI [10]. Ongoing NorDCaP projects are working on developing an AI-based tool to assist radiologists in MRI evaluation. The ambition is that the tool can improve MRI interpretation to the extent that biopsies can be avoided in men with a negative MRI. The research will at least clarify whether biopsies can be avoided with a negative MRI if the Stockholm3 risk score is below a certain cut-off.

The next step after a biopsy is a pathological examination of the tissue sample. New technologies, including sample digitization and AI, are being applied to develop a pathological tool that will provide a more reliable diagnosis of prostate biopsies [11]. With funding from NordForsk, NorDCaP aims to develop and implement a more accurate and personalized diagnostic pathway for PCa, better to deal with the clinical, health economic and ethical challenges that PCa represents today. Based on this knowledge, the consortium will develop infrastructure for a pan-Nordic quality register for PCa diagnosis. The purpose of the register is to help health care regions evaluate their own practices regarding PCa diagnosis, and to provide support for clinical implementation of best practices in the Nordic countries.

It is too early to draw conclusions, but we believe that even moderate improvements of each step in the diagnostic pathway will provide a valuable and significant improvement in the diagnosis of PCa. Ultimately, this novel and more personalized diagnostic approach should considerably reduce the number of prostate biopsies and the over-diagnosis of indolent PCa, while still ensuring early detection of clinically significant PCa when it is still curable.
